# Old Era Continues in Modern World: A Case Report of Scurvy Induced Myopathy in Patient with Chronic Alcoholism

**DOI:** 10.1155/2020/4798941

**Published:** 2020-04-09

**Authors:** Girish Singhania, Namrata Singhania, Neha Chawla

**Affiliations:** ^1^Department of Hospital Medicine, CHI St. Vincent Infirmary, Little Rock, AR, USA; ^2^Department of Hospital Medicine, Mount Carmel East Hospital, Columbus, OH, USA; ^3^Florida International University, Miami, FL, USA

## Abstract

We report a case of myopathy in a chronic alcoholic patient with scurvy who presented with generalized weakness, myalgias, and arthralgia. Our case raises awareness regarding rare interaction between vitamin C deficiency and myopathy which is seen more commonly in patients with history of chronic alcoholism and low socioeconomic status. Early treatment with vitamin C replacement is helpful in treatment of the disease and its complications.

## 1. Introduction

Ascorbic acid (vitamin C) is an essential dietary nutrient in all primates. Deficiency of vitamin C also known as scurvy is largely due to impaired collagen synthesis and disordered connective tissue. The diagnosis can be made clinically, based on a history of insufficient vitamin C intake and typical clinical signs and symptoms. The most specific symptoms are follicular hyperkeratosis and perifollicular hemorrhage, with petechiae and coiled hairs which can happen as early as 3 months after insufficient dietary intake [1]. Other symptoms include ecchymoses, bleeding, and receding gums as in gingivitis, arthralgias, anemia, and impaired wound healing [1]. The hemorrhagic skin lesions are initially flat but may coalesce and become palpable, especially on the lower extremities. Musculoskeletal pain may be caused by hemorrhage into the muscles or periosteum. A limp may be the presenting symptom, particularly in nonverbal children [2]. Generalized systemic symptoms are weakness, malaise, arthralgias, anorexia, depression, neuropathy, and vasomotor instability [3]. Cardiorespiratory symptoms, including dyspnea and hypotension, can be associated with impaired vasomotor response. Myopathy in scurvy is a rare manifestation described in literature. Although decreased in incidence, scurvy is still reported periodically in developed countries like United States of America. The most common risk factors described are alcoholism, low socioeconomic status, and psychiatric illness leading to poor oral intake. Concomitant other vitamin deficiency makes scurvy diagnosis a clinical challenge.

## 2. Case Presentation

A 55-year-old male with history of chronic heavy alcohol use and poor nutritional intake presented to the hospital for progressively worsening weakness, generalized bodyache with myalgia, arthralgia, and paresthesia in bilateral lower extremities for a year. One week prior to admission, he was unable to walk and started developing paresthesia in hands as well. He was seen by a neurologist as outpatient, and workup had not revealed any significant cause of his symptoms. His social history was positive for chronic heavy alcohol consumption. He did not have any chest pain, shortness of breath, bladder complaints, abdominal pain, or change in bowel habits. His blood pressure was 112/74 mmHg, and pulse, respiratory rate, temperature, and oxygen saturation were normal. Examination revealed sunken eyes and had lost all teeth and reported worsening of his occasional gum bleeding. He had diffuse muscle tenderness, decreased muscle strength in both upper and lower extremities, decreased pinprick, and fine touch sensation from toes up to his knees bilaterally, but sensations in his upper extremities were intact.

Initial laboratory indices like serum electrolytes, kidney function tests, and hepatic function panel were negative. Infectious workup in the form of hepatitis panel, HIV, and syphilis were negative. Inflammatory markers like erythrocyte sedimentation rate and C-reactive protein, vitamin B12, creatine kinase level, thyroid stimulating hormone, and serum protein electrophoresis were also normal/negative. Extensive workup also failed to establish the cause for muscle weakness/myopathy. MRI of head and CT scan of head were normal. Minerals level like zinc, magnesium, copper, lead, and phosphorus were normal. He was initially treated with supportive care, intravenous hydration, and multivitamins. His muscle weakness and neuropathy were attributed to chronic alcoholism at first. Later his nutritional workup revealed severe vitamin C deficiency with mild vitamin D deficiency. Vitamin C level was less than 0.1 mg/dL (normal range, 0.3 to 1.9 mg/dL), and diagnosis of scurvy was made. He was started on high dose of 1 gm/day of vitamin C. The patient showed the signs of improvement and his muscle weakness started to improve. On admission, he was unable to walk even with the support, and one week after the start of vitamin C supplementation, he started walking with the help of walker. He began working with physical therapy for strength training and was discharged to inpatient rehabilitation.

## 3. Discussion

In developed countries, vitamin C deficiency occurs mostly in severely malnourished individuals, drug and alcohol abusers, or those living in poverty or on diets devoid of fruits and vegetables [4, 5]. In the elderly or chronically ill patients, scurvy can be seen due to their poor dietary intake [6]. Scurvy has also been described in children with autism spectrum disorder who do not eat fruits and vegetable [2, 7, 8]. It is also seen in patients with iron overload due to frequent blood transfusions in diseases like sickle cell disease or thalassemia, or a history of bone marrow transplantation [9]. Iron overload can precipitate scurvy because ferric deposits accelerate the catabolism of ascorbic acid [10].

Symptoms of scurvy are usually seen when the plasma concentration of vitamin C is less than 0.2 mg/dL (11 micromol/L) [11]. Common symptoms include petechiae, ecchymoses, hyperkeratosis, perifollicular hemorrhage, and gingival swelling. In severe cases, it can cause muscle weakness. In a study, it was found that scurvy can cause upregulation of muscle atrophy related genes like forkhead box O1 (FOXO1), atrogin1/muscle atrophy F-box (MAFbx), and others [12]. The authors also found that after replacement with vitamin C the expression of these genes decreases [12]. The treatment for scurvy is vitamin C supplementation and reversal of the conditions that led to its deficiency. A wide range of replacement doses have been used successfully. For children, recommended initial doses are 100 mg ascorbic acid given three times daily for one week, then once a day for several weeks until the symptoms are fully recovered. Recommended doses for adults are usually between 300 to 1000 mg daily for one month [13]. Once the appropriate therapy is started, symptoms start to improve as early as 24 hours of treatment; bruising and gingival bleeding resolve within a few weeks.

We report this case of scurvy with its rare manifestation of myopathy to acknowledge the variable symptomatology and clinical presentation of scurvy. Clinicians should have higher index of suspicion for scurvy in the clinical presence of alcohol abuse and other risk factors. Failure to diagnose this disease can potentially lead to extensive and expensive medical tests, as well as missing a simple treatment that can prevent further complications.
